# Bacterial Virulence and Prevention for Human Spaceflight

**DOI:** 10.3390/life13030656

**Published:** 2023-02-27

**Authors:** Hakim Ullah Wazir, Pooja Narang, Giulia Silvani, Christine Mehner, Kate Poole, Catherine Burke, Joshua Chou

**Affiliations:** 1 School of Biomedical Engineering, Faculty of Engineering and Information Technology, University of Technology Sydney, Ultimo, NSW 2007, Australia; 2 School of Life Sciences, Faculty of Science, University of Technology Sydney, Ultimo, NSW 2007, Australia; 3 School of Material Science and Engineering, University of New South Wales, Sydney, NSW 2052, Australia; 4 Department of Physiology and Biomedical Engineering, Mayo Clinic, Jacksonville, FL 32224, USA; 5 EMBL Australia Node in Single Molecule Science, Faculty of Medicine, School of Medical Sciences, University of New South Wales, Sydney, NSW 2052, Australia

**Keywords:** microgravity, *S. aureus*, bacteria, microbiome, space, gentamicin, antibacterial coating, cell viability, proliferation, virulence

## Abstract

With the advancement in reusable rocket propulsion technology, space tourist trips into outer space are now becoming a possibility at a cost-effective rate. As such, astronauts will face a host of health-related challenges, particularly on long-duration space missions where maintaining a balanced healthy microbiome is going to be vital for human survival in space exploration as well as mission success. The human microbiome involves a whole list of micro-organisms that reside in and on the human host, and plays an integral role in keeping the human host healthy. However, imbalances in the microbiome have been directly linked to many human diseases. Research findings have clearly shown that the outer space environment can directly affect the normal microbiome of astronauts when the astronaut is exposed to the microgravity environment. In this study, we show that the simulation of microgravity on earth can mimic the outer space microgravity environment. *Staphylococus aureus* (*S. aureus*) was chosen for this study as it is an opportunistic pathogen, which is part of the normal human skin microflora and the nasal passages. This study’s results show that *S. aureus* proliferation was significantly increased under a microgravity environment compared to Earth’s gravity conditions, which complements previous work performed on bacteria in the outer space environment in the International Space Station (ISS). This demonstrates that this technology can be utilised here on Earth to mimic the outer space environment and to study challenging health-related questions. This in return saves us the cost on conducting experiments in the ISS and can help advance knowledge at a faster rate and produce countermeasures to mitigate the negative side effects of the hostile outer space environment on humans.

## 1. Introduction

Ever since the Apollo lunar program ended in 1972, human space exploration has been limited to a low-Earth orbit, where numerous countries participate in conducting research on the International Space Station (ISS) [1]. The recent technological advancement in reusable rockets that was successfully achieved by SpaceX has made it more possible for commercial space travel at a cost-effective rate than ever before and opens a new era of human space travel. Continued space travel appears inevitable, yet great challenges remain, and many unsolved questions, including reduced immunity and susceptibility to bacterial or virulent infections in the outer space environment. Henceforth, tools such as random positioning machine (RPM) technology can be utilised to mimic microgravity here on Earth. In this study, bacteria such as *S. aureus* are studied in response to the simulated microgravity environment created by RPM to complement the ISS studies to see if they show the same results, and, thereby, to establish RPM as a valid tool to use to study the bacterial cellular response to the microgravity environment here on Earth.

Overall, spaceflights have significant effects on the drastic shift in the immune system of astronauts. The outer space environment, including microgravity [2], radiation [3], and diet [4], has direct effects on human physiology that can be a direct health risk to astronauts, particularly during extended periods of time spent in outer space missions. Amongst these factors, the absence of gravitational force has a significant impact on the behaviour, virulence, and gene expression of micro-organisms [5,6]. Studies have shown that during spaceflights, pathogenic or opportunistic microbes, such as *S. aureus*, *Clostridium*, *E. coli*, or *P. aeruginosa*, significantly increase in their population [7,8,9,10]. This shows that the outer space environment directly impacts the behaviour of bacteria that may become harmful to astronauts during long space missions. Understanding the mechanism by which microgravity changes the integrity of bacteria that may become unfavourable to astronauts in outer space missions will help identify countermeasures to provide safer and healthier conditions for astronauts in future long-term space exploration missions. Several studies have identified an increase in bacterial virulence with decreased susceptibility to antibiotics under a microgravity environment compared to Earth’s gravity conditions [11,12,13].

In humans, *Staphylococcus aureus* (*S. aureus)* is a member of the normal skin microflora as well as the nasal passages, and it is a facultative, anaerobic, Gram-positive bacterium [14,15]. *S. aureus* has a range of complex virulence factors and regulators that provide this bacterium with the ability to transition between commensalism to a pathogenic state, as it can escape from the host immune defences. Furthermore, *S. aureus* has the ability to overcome and acquire antibiotic resistance traits through various mechanisms that are often poorly understood [16]. Hence, *S. aureus* is responsible for the significant morbidity and mortality worldwide [17]. In this study, *S. aureus* was subjected to the effects of simulated microgravity conditions for 24 h to study the cell’s viability and proliferative nature under a simulated microgravity environment compared to Earth’s gravity conditions. RPM technology is widely accepted to simulate and recreate the microgravity condition by an acceleration in the X and Y axis at a predetermined rate that neglects the Z axis (gravity). It generates randomness by using special moving patterns, also known as ‘random walk patterns’, which is advantageous in the sense that the rotation does not expose a predictable motion environment to the specimen as some living organisms have the capability to adapt to such a predictable motion pattern [18,19]. *S. aureus* was also subjected to gentamicin, an aminoglycoside antibiotic that inhibits bacterial protein synthesis [20], to determine the effects on *S. aureus* within Earth’s gravity conditions compared to the simulated microgravity conditions. Furthermore, *S. aureus* was also tested against ‘Zoono’, a commercial antibacterial product that is widely used as a surface sanitiser. Overall, this study seeks to demonstrate and validate the feasibility of an RPM simulated microgravity technology for future bacterial studies and the investigation and characterization on the efficacy of antibacterial coatings on *S. aureus* under simulated microgravity.

## 2. Materials and Methods

### 2.1. S. aureus Culture and Maintenance

*Staphylococcus aureus* (*S. aureus*, NCTC 8325-4) cells were used in this study and grown in bacterial tryptone soya broth (TSB) medium prepared by transferring 15 g (g) of tryptone soy broth (Dehydrated) powder (OXOID, Basingstoke, UK) into 500 mL of distilled water and autoclaved for sterilisation. *S. aureus* was inoculated in 10 mL TSB medium overnight at 37 °C at 200 RPM using an orbital shaker (Bioline, Eveleigh, Australia). The absorbance reading based on turbidity was taken using Infinite 200Pro microplate reader (Tecan, Männedorf, Switzerland) from the overnight inoculum at an optical density (OD) 600 nm. The overnight inoculum was diluted into 1:100 TBS medium, which was further grown for 2 h at 37 °C with orbital shaking at 200 RPM. Following the 2 h growth period, the inoculum was again further diluted into 1:100 in a T25 cell culture flask with canted neck (Product no. 430639, Corning, NY, USA) containing 70 mL (filled to maximum volume, ensuring no bubbles were present) of TSB medium for *S. aureus*. Individual T25 flasks were prepared for static control and simulated microgravity conditions alongside their blank T25 flasks consisting of TSB medium without bacteria. All the T25 flasks were incubated at 37 °C with 5% CO_2_, including the ones placed in the simulated microgravity machine. The RPM simulated microgravity technology was set up inside an incubator at 37 °C with 5% CO_2_. The RPM instrument allows for continuous random change in orientation relative to the gravity vector, resulting in an average zero gravity vector over time. The growth curve for *S. aureus* was monitored by taking the absorbance readings at OD 600nm at 0 h, 2 h, 3 h, 4 h, 5 h, 6 h, 20 h, 22 h, and 24 h. 

*Staphylococcus aureus* (*S. aureus*, NCTC 8325-4) cells were used in this study and grown in bacterial tryptone soya broth (TSB) medium prepared by transferring 15 g (g) of tryptone soy broth (Dehydrated) powder (OXOID, Basingstoke, UK) into 500 mL of distilled water and autoclaved for sterilisation. *S. aureus* was inoculated in 10 mL TSB medium overnight at 37 °C at 200 RPM using an orbital shaker (Bioline, Eveleigh, Australia). The absorbance reading based on turbidity was taken using Infinite 200Pro microplate reader (Tecan, Männedorf, Switzerland) from the overnight inoculum at an optical density (OD) 600 nm. The overnight inoculum was diluted into 1:100 TBS medium, which was further grown for 2 h at 37 °C with orbital shaking at 200 RPM. Following the 2 h growth period, the inoculum was again further diluted into 1:100 in a T25 cell culture flask with canted neck (Product no. 430639, Corning, NY, USA) containing 70 mL (filled to maximum volume, ensuring no bubbles were present) of TSB medium for *S. aureus*. Individual T25 flasks were prepared for static control and simulated microgravity conditions alongside their blank T25 flasks consisting of TSB medium without bacteria. All the T25 flasks were incubated at 37 °C with 5% CO_2_, including the ones placed in the simulated microgravity machine. The RPM simulated microgravity technology was set up inside an incubator at 37 °C with 5% CO_2_. The RPM instrument allows for continuous random change in orientation relative to the gravity vector, resulting in an average zero gravity vector over time. The growth curve for *S. aureus* was monitored by taking the absorbance readings at OD 600nm at 0 h, 2 h, 3 h, 4 h, 5 h, 6 h, 20 h, 22 h, and 24 h. 

### 2.2. Antibiotic Treatment on S. aureus 

Gentamicin sulphate (Sigma-Aldrich, Burlington, MA, USA) at 1 mg/mL was tested on *S. aureus* response to determine gentamicin effectiveness in gravity and simulated microgravity conditions. *S. aureus* was cultured and maintained as described above with the TSB medium supplemented with and without gentamicin sulphate at 1 mg/mL, followed by introducing *S. aureus* at 1:100 dilution. The absorbance readings for *S. aureus* at OD 600 nm were taken at 0 h and at 24 h using Infinite 200Pro microplate reader.

### 2.3. Antibacterial Coated Flask

Antibacterial coating was used to determine the effectiveness against bacteria under gravity and simulated microgravity conditions. The T25 flask was first sprayed into with the sanitiser (Zoono, Sydney, Australia), a commercialised antibacterial spray, and left drying for 15 min before filling it with 70 mL of TSB medium. Following bacterial growth and maintenance conditions, previously explained *S. aureus* were introduced at 1:100 dilution into a T25 flask containing 70 mL TSB medium and treated with and without the antibacterial surface sanitiser. The T25 flasks were prepared for static control and simulated microgravity conditions. The absorbance readings for *S. aureus* at OD 600 nm were taken at 0 h and at 24 h using Infinite 200Pro microplate reader. 

### 2.4. Cell Viability Analysis of S. aureus 

After 24 h of *S. aureus* cell culture, the cell viability for both bacteria under the gravity and simulated microgravity conditions were analysed in accordance with the manufacturer’s instructions with the PrestoBlue cell viability reagent (ThermoFisher Scientific, Waltham, MA, USA). PrestoBlue is a resazurin-based solution that allows for live cells to be monitored and detected within a cell culture medium. Briefly, at the end of 24 h for each set of experiments including growth curve, with and without gentamicin sulphate and with and without antibacterial coating, the cells were incubated with the PrestoBlue solution for 10 min at 37 °C. After 10 min, the fluorescence intensity at an excitation wavelength of 560 nm and emission wavelength of 590 nm were measured using the Infinite 200Pro microplate reader (Tecan, Männedorf, Switzerland). 

### 2.5. Live/Dead Analysis of S. aureus

To evaluate viability of *S. aureus,* live/dead assay using BacLight Bacterial Viability Kit (ThermoFisher Scientific, USA) was performed. Images were captured using DeltaVision Elite Deconvolution Microscope equipped with the 100× (Olympus 100×/1.40) objective lens using the green fluorescent (FITC) channels, and the dead bacterial cells images were taken using the red fluorescent (mCherry) channels. The imaging settings were as follows: LED illumination for FITC was set at emission wavelength 525 nm and excitation wavelength at 475 nm, and mCherry was set at emission wavelength 625 nm and excitation wavelength at 578 nm. A total of 3 images per sample were acquired at random from each of the 3 biological replicates. 

### 2.6. Image Analysis

All image analyses, including live/dead cell count and *S. aureus* morphological changes, e.g., surface area, circularity, solidity, and aspect ratio, were performed in FIJI software. Briefly, circularity was determined by the formula: 4π × [(Area)/([perimeter]2)] where a value of 1 indicates a perfect circle and any value less than one indicates all other irregular shapes [17]. Aspect ratio (AR) is calculated using the equation AR = length of the longest chord/width of the longest chord; a value greater than 1 represents an elongated cell [17]. To obtain the total live/dead *S. aureus* cell counts per image, the protocol ‘Quantification of Live/Dead Staining Using Fiji Software’ set out by Allevi [21] BRTI Life Sciences was used for this study. 

### 2.7. Statistical Analysis

Statistical analysis was performed using GraphPad Prism 7 (GraphPad Software, San Diego, CA, USA). The PrestoBlue readings were taken in triplicates for each of the 3 biological replicates. All experiments were performed in biological triplicates (n = 3). Significance was determined using two-tailed, unpaired *t*-test and Two-Way ANOVA (with Sidak’s post hoc analysis) and one-way ANOVA with a Tukey post-hoc analysis. All values are presented as mean ± SEM (standard error of the mean). *p* values less than 0.05 were considered statistically significant. 

## 3. Results

### 3.1. Significant Increase in S. aureus Proliferation under Simulated Microgravity Conditions

**Figure 1:**
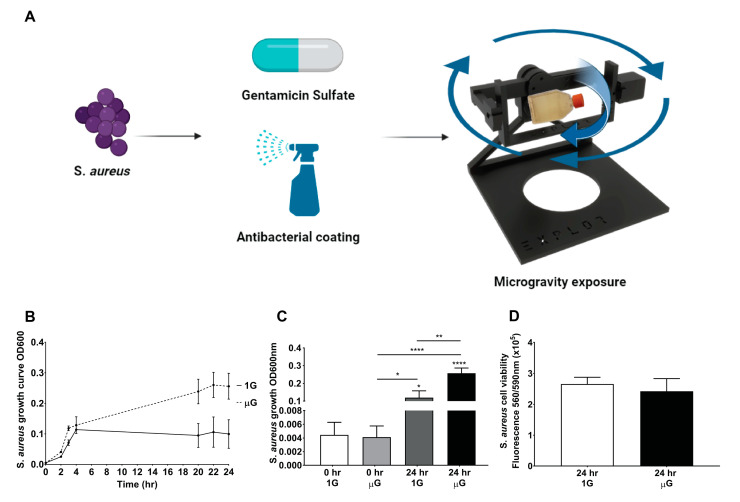
(**A**) Schematic diagram of *S. aureus* exposure to simulated microgravity environment with and without gentamicin and with and without antibacterial coating. (**B**) Growth of *S. aureus* over time under gravity and simulated microgravity conditions for 24 h (OD-600nm). (**C**) *S. aureus* growth at initial seeding density and at the end of 24 h time point. (**D**) Live *S. aureus* cells were monitored after 24 h with PrestoBlue cell viability reagent for both gravity and simulated microgravity conditions. n = 3 independent biological replicates. Data are presented as mean ± SEM. Statistical significance was determined using One-Way ANOVA (with Turkey’s post hoc analysis), two-tailed, unpaired *t*-test, and Two-Way ANOVA (with Sidak’s post hoc analysis). * *p* < 0.05, ** *p* < 0.01, **** *p* < 0.0001.

In this study, *S. aureus* was investigated in simulated microgravity conditions compared to Earth’s gravity conditions, to evaluate how *S. aureus* responds to the simulated microgravity environment as shown in the schematic diagram in Figure 1A. *S. aureus* growth was monitored over 24 h as indicated by the graph in Figure 1B,C; under gravity conditions, the exponential growth phase plateaus at 4 h, while under the simulated microgravity conditions, the exponential growth phase plateaus at 22 h. This result indicates that *S. aureus* proliferates continuously, which resulted in a 2.15 ± 0.769-fold increase for *S. aureus* under the simulated microgravity condition (OD 0.2569 ± 0.0297) compared to gravity conditions (OD 0.1197 ± 0.0386). The *S. aureus* viability analysis, shown in Figure 1D, indicates that there is no significant difference between the gravity and simulated microgravity condition, suggesting similar amounts of viable *S. aureus* are present in both the gravity and simulated microgravity conditions. 

### 3.2. S. aureus Morphology in Simulated Microgravity Is Comparable to Gravity Conditions

**Figure 2:**
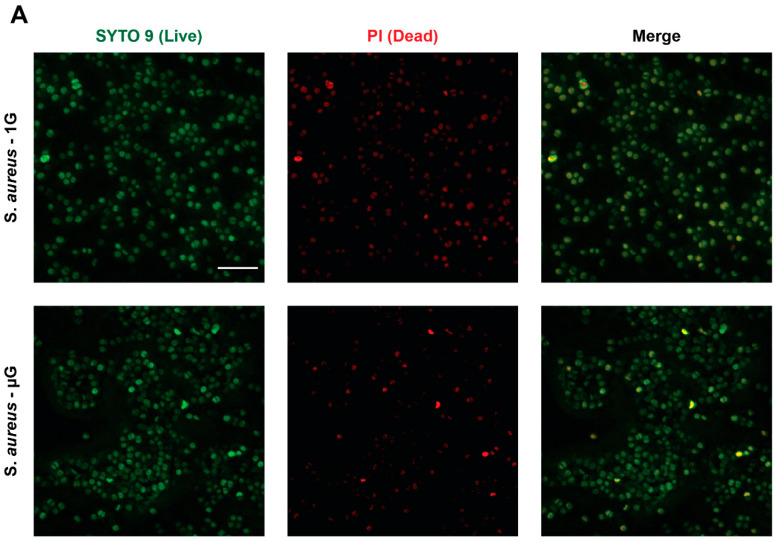

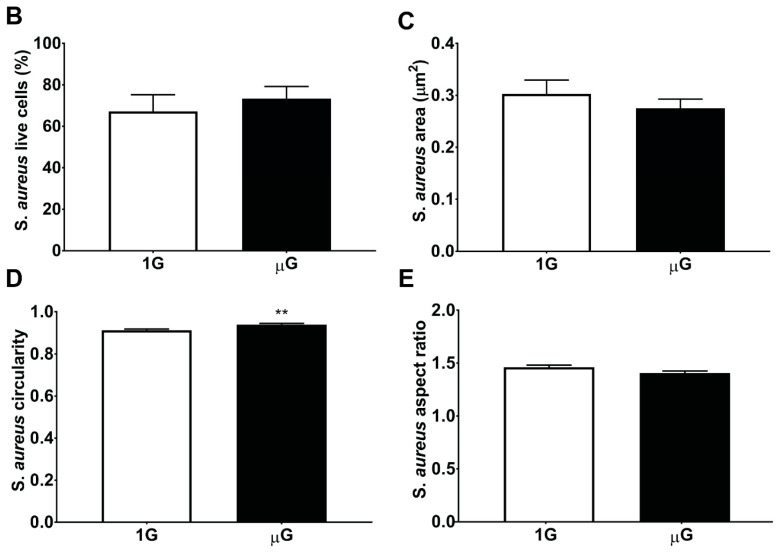
(**A**) Images of *S. aureus* stained with SYTO 9 (green; first panel) to identify live cells and propidium iodide (PI) (red; middle panel) to identify dead cells following 24 h of culture in a T25 flask under gravity conditions and simulated microgravity conditions. The last panel shows the merged images. Scale bar = 10 µm. (**B**) Percentage of *S. aureus* live cells following 24 h of culture in a T25 flask under gravity conditions and simulated microgravity conditions. (**C**) Area of *S. aureus* cells cultured in T25 flask under the mentioned conditions. (**D**) Circularity of *S. aureus* cells cultured in T25 flask under the mentioned conditions. (**E**) Aspect ratio of *S. aureus* cells cultured in T25 flask under the mentioned conditions. Data are presented as mean ± SEM. Statistical significance was determined using two-tailed, unpaired *t*-test. ** *p* < 0.01.

*S. aureus* under the gravity and simulated microgravity conditions were stained for live (green) and dead (red) (Figure 2A). The images in Figure 2A show that *S. aureus* was able to proliferate and grow in both the gravity and simulated microgravity conditions. The percentage of alive *S. aureus* as seen in Figure 2B is approximately 73.34% ± 5.838% for the simulated microgravity condition, compared to 67.22% ± 7.988% for the gravity condition, showing that there is no significant difference between the gravity and simulated microgravity conditions, suggesting that the simulated microgravity condition does not affect the survivability of *S. aureus*. An *S. aureus* morphological analysis was undertaken to examine any changes in the structural response or reorganization of the *S. aureus* cytoskeleton morphology under the simulated microgravity conditions. As shown in Figure 2C, no significant difference is observed in the surface area covered by individual *S. aureus* cells exposed to both conditions. However, there was a significant increase in circularity for *S. aureus* under the simulated microgravity conditions (Figure 2D), which suggests that the *S. aureus* shape becomes more rounded. Furthermore, this result is reinforced in the aspect ratio reported in Figure 2E, which corresponds to structural elongation, showing no significant difference between the two conditions, and the aspect ratio is closer to 1, which suggests the *S. aureus* has a rounded morphology in both the gravity and simulated microgravity conditions. 

### 3.3. Gentamicin Suppresses the Growth of S. aureus in Gravity and Simulated Microgravity Conditions

**Figure 3:**
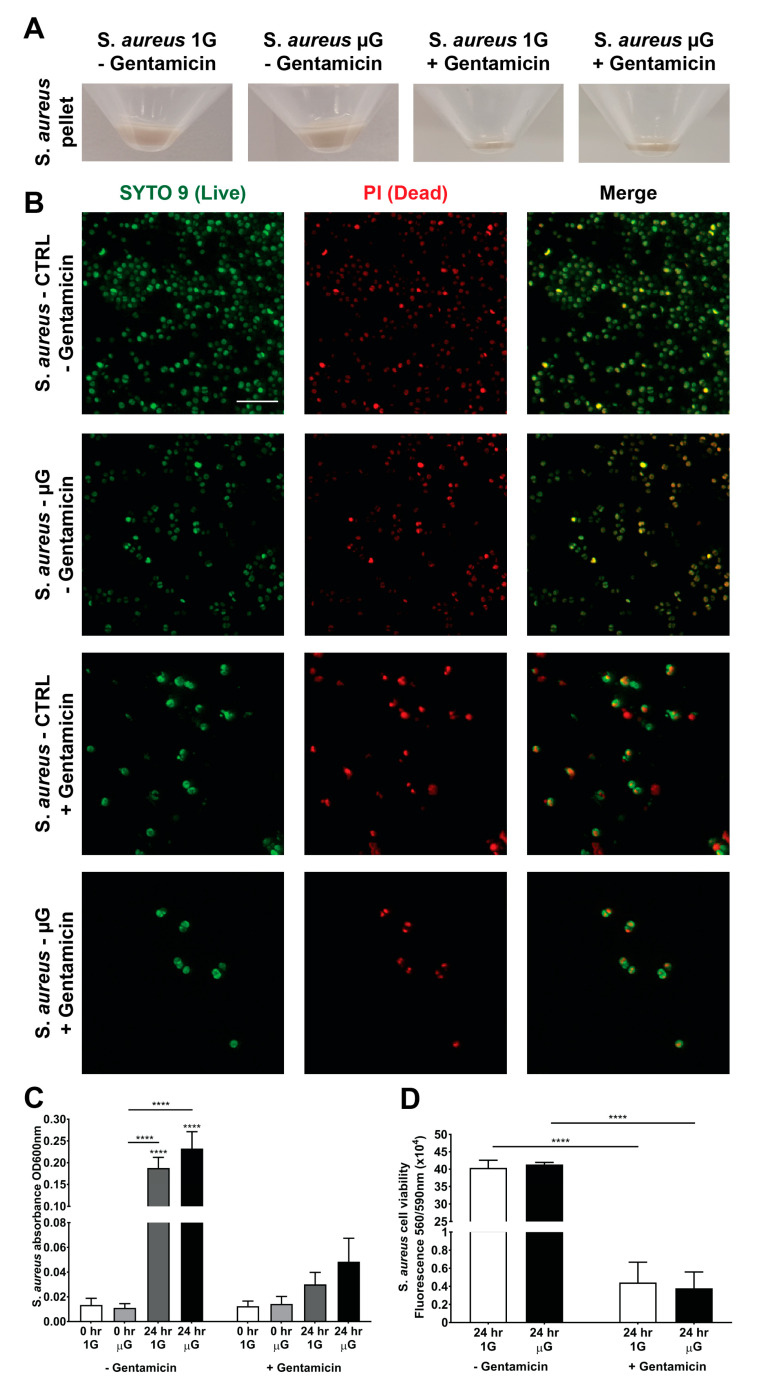
(**A**) *S. aureus* cultured in a T25 flask filled with 70 mL of TSB and with the addition of 1 mg/mL of gentamicin. Close-up images of the pellet within a 50 mL centrifuge tube were taken using a mobile phone (Samsung Galaxy S21 Ultra) after 24 h of cell culture under static control gravity conditions and simulated microgravity conditions. (**B**) Micrographs of *S. aureus* cells stained with SYTO 9 (green; first panel) to identify live cells and propidium iodide (PI) (red; middle panel) to identify dead cells following 24 h of culture in a T25 flask with 1 mg/mL of gentamicin under static control conditions and simulated microgravity conditions. The last panel shows the merged images. Scale bar = 10 µm. (**C**) *S. aureus* growth from initial seeding density and at the end of 24 h time point supplemented with and without 1 mg/mL of gentamicin (OD 600 nm) for both gravity and simulated microgravity conditions. (**D**) Live *S. aureus* cells were monitored after 24 h with PrestoBlue cell viability reagent for both gravity and simulated microgravity conditions that were supplemented with and without 1 mg/mL of gentamicin. n = 3 independent biological replicates. Data are presented as mean ± SEM. Statistical significance was determined using Two-Way ANOVA (with Sidak’s post hoc analysis). **** *p* < 0.0001.

Gentamicin is a commonly administered antibiotic for the treatment of infections including *S. aureus*. However, there are conflicting suggestions in terms of the efficacy of gentamicin under microgravity which will have significant implications for space applications. As such, this study evaluated the efficacy of gentamicin under the simulated microgravity conditions against *S. aureus* as shown in Figure 3A. The pellet of *S. aureus* was collected, and it showed a significant reduction in *S. aureus* numbers within the gentamicin group compared to the group without gentamicin. This inhibition in *S. aureus* growth was also observed through live/dead staining as indicated in Figure 3B, which shows a smaller number of *S. aureus* in the gentamicin-treated group compared to without-gentamicin treatment group. Moreover, in Figure 3C, the quantification of the *S. aureus* growth numbers shows a significant reduction in bacterial growth the with-gentamicin compared to the without-gentamicin treatment. Moreover, Figure 3D shows that gentamicin was able to effectively reduce *S. aureus* viability. Taken together, these results suggest that gentamicin remains effective, at a similar rate, in suppressing the growth of *S. aureus* after 24 h in both the gravity and simulated microgravity conditions.

### 3.4. Antibacterial Coating Inhibits the Growth of S. aureus in Simulated Microgravity

**Figure 4:**
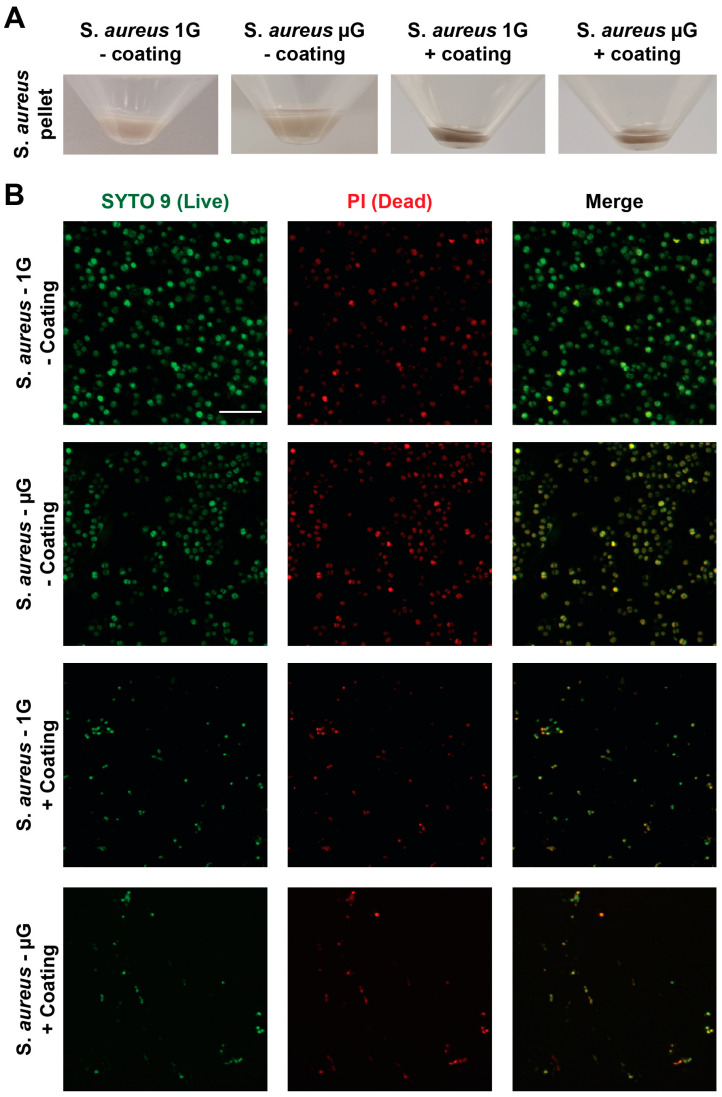

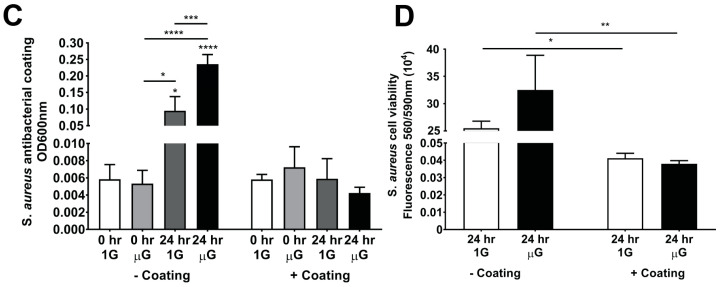
*S. aureus* cultured in a T25 flask coated with and without Zoono (antibacterial spray—commercial product) and then filled with 70 mL of TSB. (**A**) Close-up images of the pellet within a 50 mL centrifuge tube were taken using a mobile phone (Samsung Galaxy S21 Ultra) after 24 h of cell culture under static control gravity conditions and simulated microgravity conditions. (**B**) Micrographs of *S. aureus* cells stained with SYTO 9 (green; first panel) to identify live cells and propidium iodide (PI) (red; middle panel) to identify dead cells following 24 h of culture in a T25 flask coated with antibacterial spray under static control conditions and simulated microgravity conditions. The last panel shows the merged images. Scale bar = 10 µm. (**C**) *S. aureus* growth from initial seeding density and at the end of 24 h time point coated with and without Zoono (antibacterial spray) (OD 600 nm) for both gravity and simulated microgravity conditions. (**D**) Live *S. aureus* cells were monitored after 24 h with PrestoBlue cell viability reagent for both gravity and simulated microgravity conditions treated with and without Zoono antibacterial spray. n = 3 independent biological replicates. Data are presented as mean ± SEM. Statistical significance was determined using Two-Way ANOVA (with Sidak’s post hoc analysis). * *p* < 0.05, ** *p* < 0.01, *** *p* < 0.001, **** *p* < 0.0001.

To determine the effectiveness of a commercialised antibacterial product against *S. aureus* under the gravity and simulated microgravity conditions, the antibacterial spray in the form of a surface sanitiser was used to coat T25 flasks before introducing *S. aureus* into the flasks. The effectiveness of the sanitiser on *S. aureus* proliferation can be observed in Figure 4A, where the *S. aureus* antibacterial-coated samples for both the gravity and simulated microgravity conditions show a pellet after 24 h, significantly reduced with a black shaded appearance compared to the non-coated group. The live/dead cell viability stain on *S. aureus* observed in Figure 4B shows the reduced amount of *S. aureus* in the antibacterial-coated group compared to the non-coated group, suggesting that *S. aureus* proliferation is suppressed, shown by the majority of the cells being dead. Furthermore, the antibacterial coated flask seen in Figure 4C had no significant difference in *S. aureus* proliferation from the initial seeding density to the 24 h time point for both the gravity and simulated microgravity conditions. On the other hand, the non-coated flask had a significant increase in *S. aureus* proliferation for both the gravity and simulated microgravity samples after 24 h, demonstrating that the antibacterial coating is effective in suppressing/killing *S. aureus* under both the gravity and simulated microgravity conditions. *S. aureus* cell viability as seen in Figure 4D also shows that there is a significant increase of viable *S. aureus* in the non-antibacterial samples for both the gravity and simulated microgravity samples, confirming that the antibacterial coating is effective in suppressing/killing *S. aureus* under both the gravity and simulated microgravity conditions. 

## 4. Discussion

With human spaceflight being the next frontier, manned missions in space explorations for extended periods of time will bring a host of health-related challenges for astronauts. However, many challenges such as bacterial or viral infections remain unanswered. These challenges must be addressed as studies have shown bacteria becoming “super bugs” in microgravity conditions [4,22,23,24]. As such, we need to develop strategies to protect humans in space as well as upon their return, and ensure that there is no cross-contamination and that “super bugs” are not introduced into the community. This will not only be a challenge, but will also be crucial to astronauts’ health and mission success.

Different studies, conducted on the ISS [25,26,27], have shown bacteria, including *S. aureus*, having an accelerated proliferation rate under the microgravity condition, and the effects of antibiotics such as gentamicin having limited effects. To advance the fundamental understanding behind the mechanisms and functions of *S. aureus* and others alike is critical to human healthcare, as well as current and future human space exploration missions. With the emergence of “space tourism” and more launch providers to space, it is inevitable that more humans will travel into space. General healthcare issues including bacterial infections are critical issues that need to be addressed, and an effective mitigation strategy is needed. To this end, the aim of this study is to determine if the *S. aureus* increased proliferative state can be replicated here on Earth utilizing simulated microgravity equipment (EXPLOR Space Technologies, Australia), so as to ensure we can conduct more and reproducible experiments here on Earth to further the progress of preventative strategies and to examine if any existing antibacterial technologies are capable of being applied for *S. aureus* prevention. 

In this study, the results showed that simulated microgravity technology is able to achieve a 2.15 ± 0.769-fold increase in *S. aureus* proliferation within 24 h of incubation, which is similar to published studies [28,29,30], whilst maintaining the viability of the cells. This result is critical as it demonstrate the feasibility of applying simulated microgravity technology for future bacterial assays and studies and product evaluation. Live/dead staining of *S. aureus* showed similar levels in the gravity and simulated microgravity condition, and an evaluation into the bacteria morphology also showed no significant differences. Interestingly, these results suggest that the enhanced proliferation of *S. aureus* under simulated microgravity is not due to the bacterial structural sensors, while it can likely refer to other internal mechanisms and pathways related to cellular adhesion capabilities [31]. *S. aureus* is considered an opportunistic pathogen, which means it is able to adapt and proliferate under a variety of environmental conditions [32]. Studies have indicated that shear stress plays a major role in bacterial adhesion and proliferation [5,33]. The surface component ‘microbial surface components recognizing adhesive matrix molecules’ (MSCRAMMs), which are involved in recognizing adhesive matrix molecules, have been shown in the *S. aureus* binding efficiency to host factor peaks under low fluid shear stress [34,35]. A low shear stress environment has been established by NASA-designed rotating-wall vessel bioreactors that were used in spaceflights for cell culture systems. Mathematical modelling has shown that the cells within the rotating-well vessel bioreactors experience less than 1 dynes/cm^2^ of fluid shear stress [36]. Henceforth, the simulated microgravity machine utilized in this study may also expose *S. aureus* to low shear stress that directly affects the increase in *S. aureus* growth compared to the static control Earth’s gravity conditions observed in this study. 

In the event of an *S. aureus* infection, antibiotics are commonly administered as a form of treatment strategy. Whilst studies on the ISS have shown the inhibition of gentamicin efficacy [27,37,38], our results showed that gentamicin under the simulated microgravity conditions achieves a 4.8 ± 2.05 (OD600)-fold decrease compared to no gentamicin, whereas, under Earth’s gravity conditions, gentamicin achieves a 6.24 ± 2.53-fold decrease compared to no gentamicin. Thereby, gentamicin is contributing to the inhibition of *S. aureus* proliferation under both tested conditions. This result plays into the unresolved question of whether the effects studies have shown on the ISS are due to microgravity, space radiation, or a combination of both [25]. Our results would suggest that the degradation of the effectiveness of gentamicin on the ISS can very well be due to space radiation and/or combination of both. The ongoing research being undertaken by Professor Volker Hessel and his team at the University of Adelaide, whose research findings show that cosmic rays to which medicines taken on space missions are exposed limit their space shelf life, and this can even lead to the formulation of toxic substances as the drug contents degrade over time in the outer space environment a lot faster compared to Earth’s environment [39]. To further elaborate on bacteria being more resistant to antibiotics in the outer space environment as well as being more dominant in its proliferation compared to Earth’s gravity environment as shown in this study, ref. [40]’s study findings showed that bacteria such *E. coli* had 50 stress-response genes upregulated in response to the microgravity environment compared to Earth’s gravity control groups. This, in effect, would suggest that the direct stress response initiated by the microgravity environment including nutrients, starvation, and acid-shock can effectively upregulate the stress-response pathways that correlate with antibiotic stress, and, in doing so, contributes to the increased antibiotic stress tolerance, which is normally observed in bacteria within outer space environments [40]. These results again demonstrate the importance of a more widespread, reproducible, and standardized study conducted on Earth to develop precision preventative strategies. Due to the scope of this study and the limitation in accessing a radiation source, future studies will investigate the role of microgravity and/or radiation. This will help inform the type of antibiotic formulation and protection that is required for antibiotic pharmaceuticals to remain effective in space and under microgravity. The above results demonstrate the feasibility of using simulated microgravity as a model technology to study *S. aureus* and pharmaceuticals under microgravity. The usage and application of antibiotics is only required in an infection state or if critically needed. The ideal scenario is to develop preventative strategies to minimize infection and *S. aureus* prevalence in the first place. 

Since the introduction of the COVID-19 pandemic, there has been significant acceptance and advancement in bacterial and viral prevention and mitigation. Amongst these, a commercially available product ZOONO™ has been demonstrated to be highly effective in bacterial prevalence, by directly “bursting” the bacterial cell membrane. In addition, the application of the product is a simple spray on surfaces and it has an extended efficiency period of up to 30 days. This is attractive in the context of space missions, as resupply and space tourist and astronaut missions are generally within this time. Furthermore, unlike alcohol wipes/similar technology, this is non-alcohol-based and, as such, feasible for space crew. The practicality of applying a ZOONO™ coating on the crew and/or the internal space craft will ensure a layer of protection against the existing and the introduction of new bacterial exposure. Our results demonstrated that the ZOONO™ antibacterial coating can achieve a significant difference in the reduction of *S. aureus* growth in Earth’s gravity condition and under simulated microgravity. This is further supported by the *S. aureus* viability results which also demonstrated a significant reduction of viable *S. aureus* proliferation compared to not applying a ZOONO™ coating under both the tested conditions. The collected bacteria pellet showed a black/darkened state of the dead bacteria. 

In summary, this study has demonstrated the feasibility of the use of simulated microgravity technology to further advance our understanding of bacterial function and mechanisms, the study of pharmacokinetics including gentamicin, and how future studies can develop prevention technologies and the application of immediate commercial products that can be tested and applied for current and future space missions. This also reinforce the need for co-development and research between existing industries in supporting human spaceflight. It can be seen from this study that the gap in the understanding of how *S. aureus* functions, and the specific mechanosensing pathways and mechanisms on which the bacteria rely and process antibiotics remain largely unknown, and, previously, this is due to the lack of access to the microgravity environment, but as shown in this study, there are available technologies to support this type of research and product development. Future studies will investigate the underlying mechanobiological functions of how *S. aureus* responds to the microgravity environment and to validate these findings in other common bacterial strains. As human spaceflights take off, it is critical to be able to resolve issues surrounding bacterial infection and prevention. 

## Data Availability

Not Applicable.
